# Model worms: knowledge gains and risks associated with the use of model species in parasitological research

**DOI:** 10.1017/S0031182023000963

**Published:** 2023-09

**Authors:** Robert Poulin

**Affiliations:** Department of Zoology, University of Otago, Dunedin 9054, New Zealand

**Keywords:** artificial selection, evolutionary divergence, founder effect, genetic drift, *Heligmosomoides bakeri*, laboratory culture, serial passage

## Abstract

Model parasite species, whose entire life cycle can be completed in the laboratory and maintained for multiple generations, have played a fundamental role in our understanding of host–parasite interactions. Yet, keeping parasites in laboratory conditions may expose them to unnatural evolutionary pressures, and using laboratory cultures for research is therefore not without limitations. Using 2 widely-used model helminth species, the cestode *Hymenolepis diminuta* and the nematode *Heligmosomoides polygyrus*, I illustrate the caution needed when interpreting experimental results on model species. I first review more than 1200 experimental studies published on these species in the past 4 decades, to determine which research areas they have contributed to. This is followed by an examination of the institutional laboratory cultures that have provided the parasites used in these studies. Some of these have persisted for decades and accounted for a substantial proportion of published studies, whereas others have been short-lived. Using information provided by the curators of active cultures, I summarize data on their origins and maintenance conditions. Finally, I discuss how laboratory cultures may have been subject to the influence of evolutionary genetic processes, such as founder effects, genetic drift and inbreeding. I also address the possibility that serial passage through laboratory hosts across multiple generations has exerted artificial selection on several parasite traits, resulting in genetic and phenotypic divergence among laboratory cultures, and between these cultures and natural parasite populations. I conclude with recommendations for the continued usage of laboratory helminth cultures aimed at maximizing their important contribution to parasitological research.

## Graphical abstract

**Figure:**
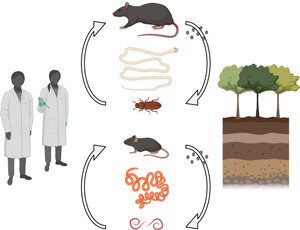

## Introduction

Model organisms have undoubtedly been fundamental resources for research progress in the biological sciences (Müller and Grossniklaus, 2010; Alfred and Baldwin, 2015). Some of the main animal model species include the nematode *Caenorhabditis elegans*, the fruit fly *Drosophila melanogaster*, the zebrafish *Danio rerio*, the clawed frog *Xenopus laevis* and the house mouse *Mus musculus*. Huge investments and efforts have made them tractable experimental models supported by established research infrastructure, standardized protocols for their laboratory maintenance and use in experiments, and state-of-the-art genomic tools. The rationale behind the focus on selected model species is to ‘learn about the general by studying the specific’ (Kellogg and Shaffer, 1993). However, a focus on model organisms leads to increasing depth of knowledge about a few species, at the expense of breadth of knowledge across many species. Peculiar aspects of the biology of model species may bias research directions and our understanding of general biological processes (Bolker, 1995, 2017). In some cases, the species that have become established models have done so because they were the best choices out of the available options, based on their biological properties; in other cases, they have done so not necessarily because they are representative of animals in general, but for either idiosyncratic reasons or because of influential researchers who first studied them. Their biology in the laboratory may not even match their biology in nature (Alfred and Baldwin, 2015). Many have argued that we need to expand the range of ‘model’ species on which we build our biological knowledge (Russell *et al.* 2017). However, despite all their associated limitations (Bolker, 1995, 2017; Katz, 2016), model species remain one of the most important research assets at our disposal for scientific advances in biology.

**Figure 1.**
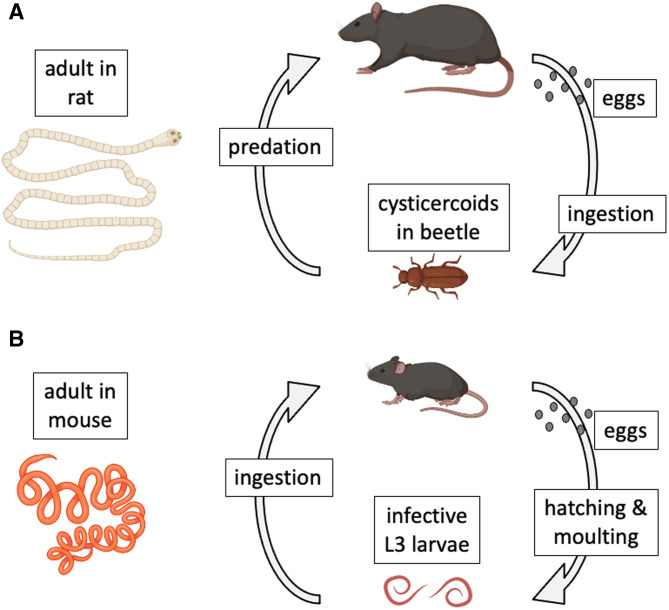
Natural life cycles of (A) the cestode *Hymenolepis diminuta* and (B) the nematode *Heligmosomoides polygyrus*. The figure uses icons from BioRender.com.

Model species have also played key roles in the study of host–parasite interactions (Poulin, 2021). In a recent review of the use of model organisms in the study of disease ecology (Vale and Duffy, 2021), 2 helminth parasite species that can be cultured in the laboratory (i.e. their entire life cycle can be completed in the laboratory and maintained for multiple generations) were identified as genuine model species. The first is the cestode *Hymenolepis diminuta* (order Cyclophyllidea). This cestode has a complex, 2-host life cycle (Rajeev *et al*., 2022). Adult worms live in the intestine of the definitive host, a role that in nature can be played by a variety of rodents across the Northern hemisphere (Fig. 1A). When gravid proglottids detach from the worm and disintegrate, eggs are released and then expelled in host feces to the outside environment. After a suitable insect intermediate host (usually a beetle) ingests an egg, an oncosphere hatches from the egg and penetrates the insect's gut wall to develop into a cysticercoid. The life cycle is completed when a suitable rodent preys on an infected insect. The worm grows rapidly within the rodent's intestine, reaching maturity after a few weeks and attaining an average length of 30 cm. *Hymenolepis diminuta* is a zoonotic cestode, as humans can also become infected if they accidentally ingest infected insects (Panti-May *et al*., 2020). The life cycle can be completed relatively easily under controlled conditions, using various strains of laboratory rats (*Rattus norvegicus*) as definitive hosts and beetles (genera *Tenebrio* or *Tribolium*) as intermediate hosts. For this reason, *H. diminuta* has become a widely used model species in parasitological research (Nowak *et al*., 2019; Sulima-Celińska *et al*., 2022).

The second model helminth species is the nematode *Heligmosomoides polygyrus* (order Rhabditida) (Johnston *et al*., 2015). It was formerly called *Nematospiroides dubius*, and is now also known as *H. bakeri*, or *H. polygyrus bakeri*, as it may consist of more than 1 species or subspecies (Behnke *et al*., 1991; Musah-Eroje *et al*., 2023; Stevens *et al*., 2023); more on this later. Given that there is no universal consensus on this matter, and that the arguments presented here do not rely on the species status or name of the worms, I will refer to them throughout the article as *H. polygyrus*, for simplicity and for consistency with most earlier publications on this parasite. The nematode has a simple 1-host life cycle and is a common parasite of many species of wild rodents across Eurasia and North America (Fig. 1B). Adult worms live in the small intestine of rodents, with female worms reaching 12–13 mm in length, about twice the size of male worms. Eggs released by females pass out in host feces. Under optimal environmental conditions, they hatch in about 24 h into L1 larvae. After a couple of moults, they develop into infective L3 larvae. When a suitable rodent ingests L3 larvae, the latter will exsheath after reaching the intestinal lumen, invade the intestinal wall where they develop into adult worms and return to the lumen about 2 weeks post-infection. The life cycle can also be completed easily in captivity, using various strains of laboratory mice (*M. musculus*) as hosts (Johnston *et al*., 2015). For this reason, just like *H. diminuta*, *H. polygyrus* has also become a popular model species in experimental parasitology (Monroy and Enriquez, 1992; Behnke *et al*., 2009; Harris *et al*., 2014).

These 2 model parasites have made huge contributions to parasitology, from casting light on the most basic aspects of helminth physiology and biochemistry, to serving as ideal candidates for tests of new anthelmintic drugs (Monroy and Enriquez, 1992; Behnke *et al*., 2009; Sulima-Celińska *et al*., 2022). The establishment of these model systems opened up research avenues that would not have been possible had they not been available. Much of our modern understanding of mammal–parasite interactions, including human–parasite interactions, is founded on what we learned from their use in the past decades. The knowledge they have yielded can be even more useful, however, if considered and interpreted in the context of some of the limitations associated with reliance on laboratory-cultured model parasites. Here, I first present a quantitative synthesis of patterns in the use of these 2 model helminth species over time and across research areas. Then, based on information either published or provided by the current users and curators of laboratory populations of the 2 model species, I summarize the main characteristics of laboratory helminth cultures with a focus on evolutionary considerations. Finally, I discuss the conditions experienced by helminths within laboratory cultures in the light of fundamental concepts from population genetics and evolutionary theory. This is followed by a re-examination of the suitability of laboratory cultures for certain kinds of research questions, given the likely genetic and phenotypic changes they have undergone since being isolated from wild populations. The goal of this scrutiny is not to discredit the huge contributions that research based on laboratory helminth cultures has made to our understanding of host–parasite interactions. Far from it, this review instead aims to provide a broader evolutionary context in which to interpret some earlier findings and gain further insights into what they reveal.

## Historical overview of model species usage in parasitological research

### Data compilation

Two ‘topic’ searches of the Web of Science database were conducted for relevant articles published between 1980 and 2022, inclusively. The first search used the string: ‘*Hymenolepis diminuta*’; whereas the second used the string: (‘*Heligmosomoides polygyrus*’ OR ‘*Heligmosomoides bakeri*’ OR ‘*Nematospiroides dubius*’). Abstracts and conference proceedings were excluded. The 1446 relevant publications (695 on *H. diminuta* and 751 on *H. polygyrus*) retrieved by each search were checked individually to separate publications into experimental laboratory studies, field studies of natural parasite populations (including surveys of contamination of environmental substrates with the parasite's eggs), case reports of human infections and literature reviews (including meta-analyses) specifically about the focal helminth species.

I recorded the year of publication of each retrieved article, as well as other basic details (authors, journal, etc.). In addition, for each experimental laboratory study, I recorded (i) the research area and therefore the parasite traits investigated (see below), (ii) the identity of the intermediate host species used in the case of *H. diminuta*, and (iii) the identity of the laboratory culture from which the helminths were sourced. In most cases, this corresponded to the institution where the research was conducted, with parasites from those cultures sometimes referred to as ‘strains’ named after their source institution. In a few cases, the authors identified another institution as the source of their specimens, though the parasites had usually been cultured at the authors' institution for a few generations prior to their first use in research; therefore, the authors' institution was still considered at the source culture populations in those cases. The identity of the laboratory culture was not always clear in the case of multi-authored studies involving several institutions; as a rule of thumb, if a laboratory that had produced multiple prior studies on the focal helminth species was involved in a more recent study, it was taken to be the home of the laboratory culture unless information in the article suggested otherwise. The full list of publications retrieved and their associated data is available as Supplementary Material (Table S1).

### Temporal patterns of model species usage

**Figure 2.**
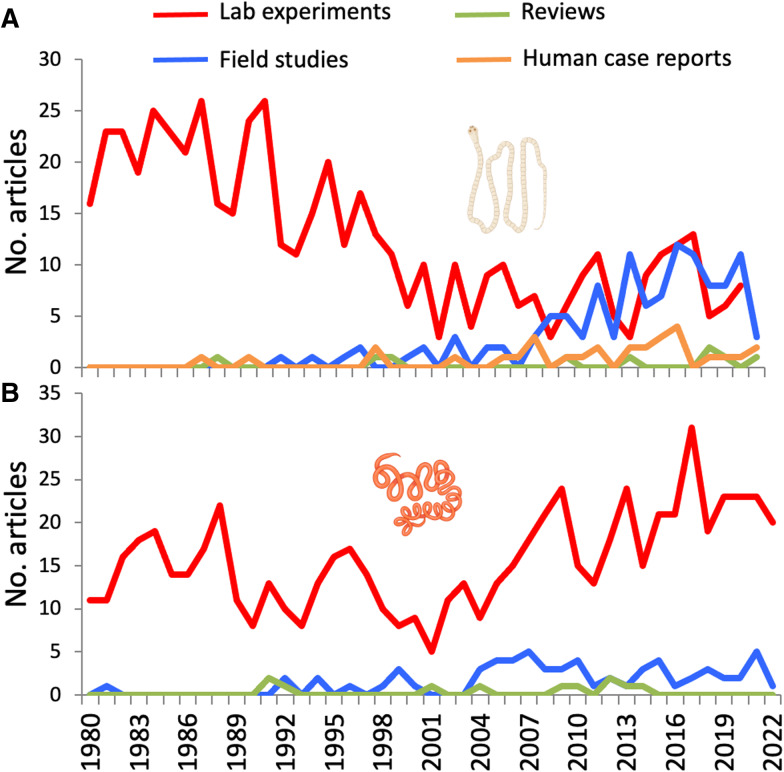
Annual numbers of articles of different types published between 1980 and 2002 concerning (A) the cestode *Hymenolepis diminuta* and (B) the nematode *Heligmosomoides polygyrus*. The figure uses icons from BioRender.com.

For *H. diminuta*, the literature search yielded 537 experimental laboratory studies, 119 field studies of natural populations, 30 case reports of human infections and 9 reviews focused on this particular cestode. The number of experimental laboratory studies published per year dropped during the 1990s and settled on about 5–10 annually, while the numbers of field studies and case reports of human infections have increased after the year 2000 (Fig. 2A). For *H. polygyrus*, the search retrieved 676 experimental laboratory studies, 64 field studies of natural populations and 11 reviews focused on this particular nematode. For this species, the number of experimental laboratory studies published per year has risen slightly since the year 2000, as have field studies (Fig. 2B). These temporal patterns are subject to some error, given the incompleteness and biases associated with the literature coverage of the Web of Science (Mongeon and Paul-Hus, 2016); however, they certainly confirm that these 2 helminth species have been, and continue to be, major model systems for parasitological research.

**Figure 3.**
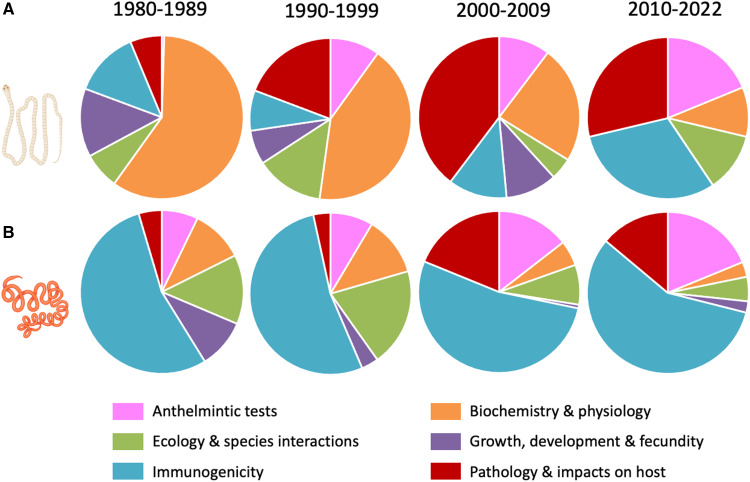
Relative proportions of experimental laboratory studies in 6 different research areas, shown separately for each of the last 4 decades, which used (A) the cestode *Hymenolepis diminuta* and (B) the nematode *Heligmosomoides polygyrus*. See the main text for a fuller description of each research area. The figure uses icons from BioRender.com.

The 1213 experimental laboratory studies (537 on *H. diminuta* and 676 on *H. polygyrus*) were separated into the following 6 categories based on the research area and/or the parasite traits investigated: (i) biochemistry, physiology or metabolism of the parasite; (ii) growth, development, morphology or fecundity of the parasite; (iii) other aspects of the biology or ecology of the parasite, including host manipulation, transmission processes, within-host intraspecific competition, etc.; (iv) pathogenicity, virulence or other impacts on host biology, health or fitness, including positive impacts on health or fitness; (v) immunogenicity or any aspect of the host's immune response against the parasite; and (vi) anthelmintic testing involving any drug or compound. There have been clear shifts in research focus over time for both model species (Fig. 3). In the case of the cestode *H. diminuta*, research on its biochemistry, physiology and metabolism went from the dominant area of investigation to a relatively minor one, while research on its impacts on host health and fitness and the immune response it induces, as well as its use in studies of anthelmintic efficacy, have increased over time. In contrast, research involving the nematode *H. polygyrus* has always been dominated by studies on its immunogenicity and various aspects of the host immune response. Studies on the pathology it induces and its response to anthelmintic compounds have increased in frequency in the 4 decades covered here, at the expense of research on its biochemistry and ecology (Fig. 3). To some extent, these temporal changes in the nature of research conducted on the 2 model species may simply reflect the waxing and waning of the careers of key scientists who used these helminths for their investigations in different subdisciplines.

**Figure 4.**
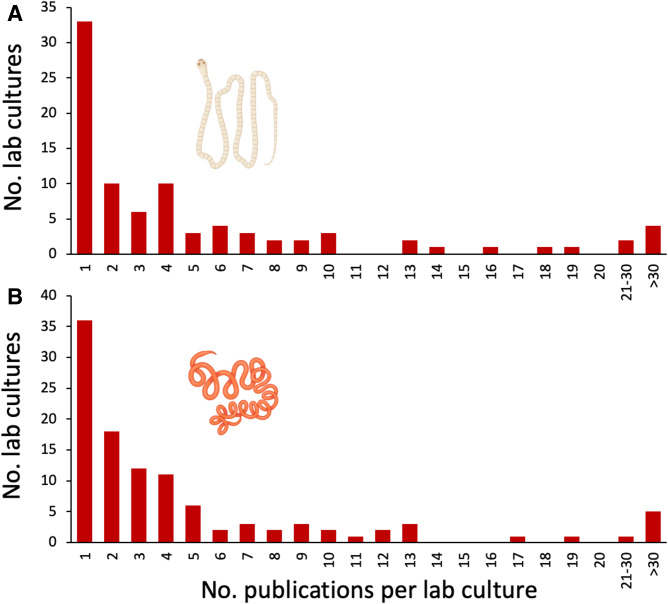
Frequency distribution of numbers of published experimental studies per institutional laboratory culture of (A) the cestode *Hymenolepis diminuta* and (B) the nematode *Heligmosomoides polygyrus*. The figure uses icons from BioRender.com.

From the information available in each experimental laboratory study, 89 distinct laboratory cultures for *H. diminuta* and 109 for *H. polygyrus* could be identified (no laboratory culture could be assigned to 4 out of 537 experimental studies on *H. diminuta* and 9 out of 676 studies on *H. polygyrus*). These cultures were spread across >30 countries on all continents except Antarctica. The different cultures did not contribute equally to research on the 2 model species, with a small number accounting for most published studies (Fig. 4). The laboratory cultures that produced only 1 or a few articles are most likely not ‘true’ cultures. They probably often represent situations where researchers obtained specimens from another institution for a one-off experiment and did not maintain the parasite in culture through several generations for the purpose of re-using them. Therefore, bars towards the left-hand side of the frequency distributions in Fig. 4 may be higher than they should be. Based on the procedures described above to assign a source culture to each experimental study, the most prolific laboratory culture for *H. diminuta* lead to 45 published articles, whereas that for *H. polygyrus* produced 80 published articles. Further information on the laboratory cultures is presented in the next section.

Among the 89 laboratory cultures of the cestode *H. diminuta*, 22 used the beetle *Tenebrio molitor* as intermediate host, 34 used *Tribolium confusum*, 2 used *Tribolium castaneum*, while the intermediate host species was not specified for the remaining 31 cultures. From the information given in the published experimental studies, it appears that some (no more than 5) cultures of *H. diminuta* may have switched intermediate host species at least once over the years.

## Characteristics of laboratory cultures of model helminths

### Data compilation

From the literature search described in the previous section, the research lifespan of each laboratory culture was calculated as the number of years between the first and last papers using it were published. Wherever possible, institutional name changes were taken into account to avoid counting twice those few cultures that were housed in institutions that changed names over time. It must be noted that the true lifespan of some cultures may be underestimated, as they may have been contributing to research prior to 1980 or may do so after 2022, the start and end dates of the period covered here. I then tested for a relationship across all cultures between the lifespan estimated here and the number of published studies based on them, for each model helminth species separately, using Spearman correlation coefficients.

I also identified all laboratory cultures of *H. diminuta* and *H. polygyrus* likely to be still ongoing (based on at least 5 studies published in the last 4 years of the dataset, i.e. 2019–2022), and sent a questionnaire to the corresponding authors and/or to the institutional address where the work was conducted (using the email for the department, school or institute available on their webpage). The questionnaire asked for the following information, where available: year in which the laboratory culture was established; the source of the original specimens; how many individual worms made up the founding population; the estimated number of parasite generations since the foundation; whether the population was ‘refreshed genetically’ by the addition of new individuals from an outside source, and if so how many, from where and when; how are the rodent hosts infected; and in the case of the cestode *H. diminuta*, what species of beetle intermediate host is used and how are they infected. The full list of laboratory cultures for which responses to the questionnaires were obtained and their associated data are available in Tables 1 and 2.

**Table 1.** Characteristics of a selection of laboratory cultures of the cestode *Hymenolepis diminuta*

Institution (strain)	Year of establishment	Size and source of founding population	Number of generations up to 2022	New individuals added? (year, number, source)	Intermediate host used	Method of rat infection	Method of beetle infection
Czech University of Life Sciences Prague	2010	≈3–5 adult worms (Medical University of Warsaw)	≈7–10	No	*Tribolium confusum* and *Tenebrio molitor*	Cysticercoids given *via* oral gavage, or mixed with food	Beetles in Petri dishes allowed to feed on concentrated eggs extracted from rat feces, or directly on feces of infected rats
Medical University of Warsaw (WMSil1)	1975	1 adult worm (from another unknown lab culture)	≈35^a^	No	*Tenebrio molitor*	Cysticercoids mixed with food	Beetles in Petri dishes allowed to feed on gravid proglottids, on eggs mixed with food, or directly on feces of infected rats
Medical University of Warsaw (WRShs1)	2014	1 adult worm (from an infected child)	≈5	No	*Tenebrio molitor*	Cysticercoids given *via* oral gavage	Beetles in Petri dishes allowed to feed on gravid proglottids, on eggs mixed with food, or directly on feces of infected rats
University of Georgia	2013	≈50 cysticercoids (Carolina Biological Supply Co., USA)	≈15	No	*Tenebrio molitor*	Cysticercoids given *via* oral gavage	Beetles allowed to feed on fresh gravid proglottids mixed with food
University of Alberta	ca. 1960	Unknown (Rice University)	≈50^b^	Yes (1989, number unknown, from Carolina Biological Supply Co., USA)	*Tribolium confusum*	Cysticercoids given *via* oral gavage	Beetles in Petri dishes allowed to feed on concentrated eggs extracted from rat feces
North-Eastern Hill University	2003	Unknown (Visva-Bharati University)	≈80	No	*Tribolium confusum*	Cysticercoids given *via* oral gavage	Beetles allowed to feed on eggs extracted from rat feces and mixed with flour
Visva-Bharati University	2007	≈8–12 adult worms (Gifu University)	≈90	No	*Tribolium castaneum*	Cysticercoids given *via* oral gavage	Beetles allowed to feed on fresh gravid proglottids

The information is based on the recollections and best estimates from the current keepers of the cultures.

a Until 2020 only.

b Until 2016 only.

**Table 2.** Characteristics of a selection of laboratory cultures of the nematode *Heligmosomoides polygyrus*

Institution	Year of establishment	Size and source of founding population	Number of generations up to 2022	New individuals added? (year, number, source)	Method of mouse infection
University of Dundee	2021	≈20 000 larvae (University of Glasgow)	5	No	Oral gavage of infective L3 larvae obtained from coproculture
University of Glasgow	2002	≈5000 larvae (University of Nottingham)	≈400	No	Oral gavage of infective L3 larvae obtained from coproculture
University of Nottingham	1975	Unknown (Wellcome Laboratories)	≈75–100^a^	No	Oral gavage of infective L3 larvae obtained from coproculture
Francis Crick Institute	2017	≈1000 (other lab at same institute)	≈20	Yes (2018, ≈1000, from University of Glasgow)	Oral gavage of infective L3 larvae obtained from coproculture
Freie Universität Berlin	2002	≈1000–1500 (University of Warsaw)	≈60	No	Oral gavage of infective L3 larvae obtained from coproculture
University of Warsaw	1990	≈500 larvae (University of Nottingham)	≈160	No	Oral gavage of infective L3 larvae obtained from coproculture
Swiss Tropical and Public Health Institute	2012	≈2000 larvae (University of Nottingham)	≈44	No	Oral gavage of infective L3 larvae obtained from coproculture
Université de Bourgogne	2012	≈5000 larvae (University of Edinburgh)	≈40	No	Oral gavage of infective L3 larvae obtained from coproculture
McGill University's Parasitology Institute	1983	≈600 larvae (University of Toronto)	≈140	Yes (mid-1990s, number unknown, from Rutgers University)	Oral gavage of infective L3 larvae obtained from coproculture
McGill University's Health Centre	2012	≈400–600 larvae (Trudeau Institute, New York)	≈30	Yes (2015, ≈400, from McGill University's Parasitology Institute)	Oral gavage of infective L3 larvae obtained from coproculture
USDA Beltsville Agricultural Research Center	mid-1980s	≈2000 larvae (Royal Veterinary and Agricultural University, Copenhagen)	≈200–280	No	Oral gavage of infective L3 larvae obtained from coproculture
Rutgers New Jersey Medical School	2010^b^	≈5000–7000 larvae (Beltsville Research Centre, USDA)	Unknown	No	Oral gavage of infective L3 larvae obtained from coproculture

The information is based on the recollections and best estimates from the current keepers of the cultures.

a Until 2012 only.

b Last batch of about a thousand larvae obtained from USDA in 2010, independent culture since then.

### Origins and maintenance of laboratory cultures

There are no available and detailed records tracing the origins of all laboratory cultures of the 2 helminth species, with details of their source population, the time at which they were established, etc. For the cestode *H. diminuta*, several cultures appear to have been initiated with material provided either by older institutional cultures used in research (e.g. from Rice University, Texas, USA) or by commercial suppliers (e.g. Carolina Biological Supply Company, North Carolina, USA). For *H. polygyrus*, a partial ‘family tree’ of cultures emerges, with the Wellcome Research Laboratories (UK) and the Ayerst Research Laboratory and Merck & Co. (USA) identified as the sources of some of the oldest cultures, and with the University of Nottingham (UK) and McGill University (Canada) then providing the specimens that started several more recent cultures (see Musah-Eroje *et al*., 2023). However, too little information is available on cultures of both helminths to construct a meaningful and reliable tree of relationships among cultures, with the origins and year in which founding specimens were obtained being rarely indicated.

**Figure 5.**
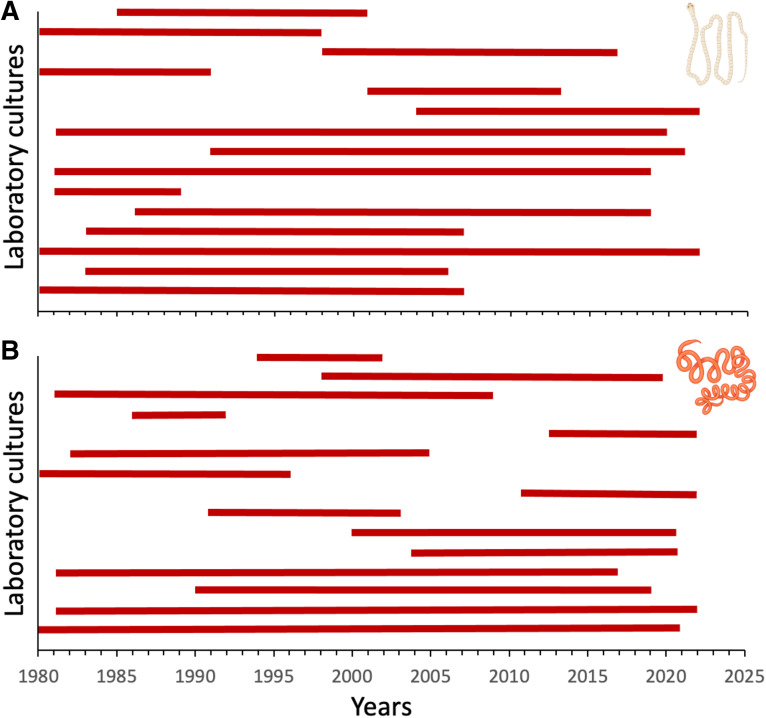
Lifespan of institutional laboratory cultures, based on the earliest and latest years of publications attributed to them, for (A) the cestode *Hymenolepis diminuta* and (B) the nematode *Heligmosomoides polygyrus*. Only the 15 most prolific cultures (i.e. those from which more articles were published) identified in the literature search are included here, shown from most (bottom) to least (top) prolific. The figure uses icons from BioRender.com.

**Figure 6.**
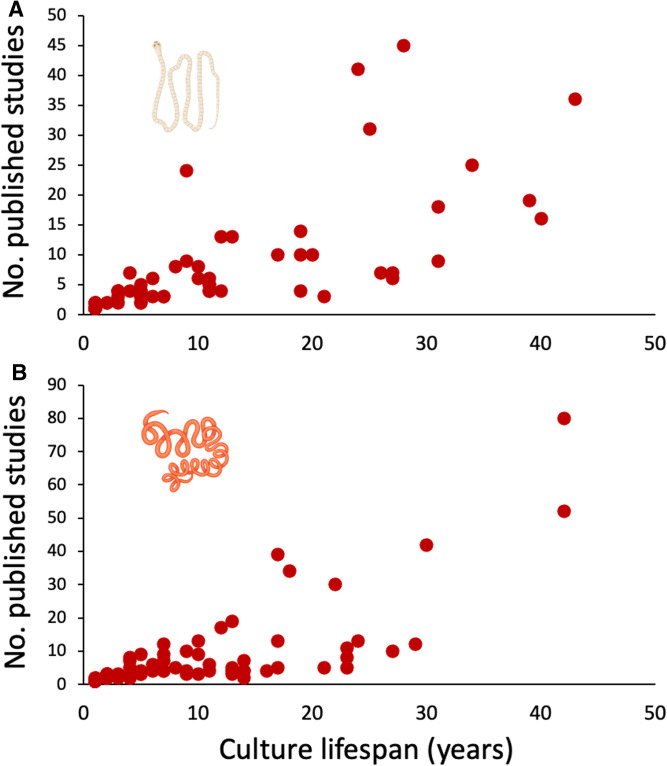
Number of published experimental studies per laboratory culture plotted against the lifespan of the culture, across (A) 89 cultures of the cestode *Hymenolepis diminuta* and (B) 109 cultures of the nematode *Heligmosomoides polygyrus*. Note that several points are stacked at the coordinate 1 year × 1 article. The figure uses icons from BioRender.com.

Across all laboratory cultures identified in the literature search, their average lifespan (with caveats mentioned earlier, i.e. based solely on Web of Science records) as sources of experimental subjects was 8.5 years (range 1–43 years) for *H. diminuta* and 7.1 years (range 1–42 years) for *H. polygyrus*. Some laboratory cultures were active only in the first part of the 4-decade period covered by the present review, some only in the second part and others throughout the whole period (Fig. 5). The most likely reasons for laboratory cultures being discontinued are probably funding cuts, tightening ethical constraints around the housing of rodents and the retirement of the main researcher using the culture. There was a highly significant positive correlation between the number of years a culture was active and the total number of published experimental studies that used it between 1980 and 2022 inclusively, for each of the 2 helminth species (*H. diminuta*: *r*_s_ = 0.929, *N* = 89, *P* < 0.0001; *H. polygyrus*: *r*_s_ = 0.925, *N* = 109, *P* < 0.0001). Nevertheless, there were many laboratory cultures identified here (again, with the caveats mentioned earlier) that did not last very long and produced very few published studies (Fig. 6).

Out of all laboratory cultures identified as still used for research purposes in recent years, responses to questionnaires were obtained for 7 cultures of *H. diminuta* and 12 cultures of *H. polygyrus* (Tables 1 and 2). When known, the number of individual worms used as founders of *H. diminuta* cultures was generally quite small, e.g. <10, as the goal was often to create an inbred strain of the parasite. The cestode cultures were maintained for between 5 and nearly 100 generations, and were generally not refreshed by the addition of new individuals from an outside source (Table 1). Rats are almost invariably infected by gavage, i.e. the cystacanths removed from infected beetles are administered orally through a blunt-tipped needle or a tube down the rat's throat. In turn, beetles are generally infected by starving them for a few days and then allowing them to feed on gravid proglottids or eggs extracted from rat feces (Table 1). In contrast, laboratory cultures of *H. polygyrus* were generally started with more individuals, although the number of infective L3 larvae used as founders to infect the first batch of mice was often 1000 or less (with a typical dose of 200 larvae per mouse). Of course, only a fraction of L3 larvae given to mice grew into adult worms, therefore the true number of genetic founders was always much smaller. The nematode cultures were maintained for between 5 and 400 generations, and were also rarely refreshed by the addition of new individuals from an external source (Table 2). Oral gavage is the method used to infect mice in all cultures, using L3 larvae hatched and grown in coproculture from host feces.

## The genetics of laboratory helminth cultures

By their very nature, laboratory populations of model organisms may be subject to a range of evolutionary processes that together contribute to create genetic differences between laboratory populations and natural ones, as well as differences among distinct laboratory populations. These processes are well-established principles in evolutionary genetics (Maynard Smith, 1998; Hartl and Clarke, 2006). Over time, these differences can become more pronounced, to the point that (i) laboratory populations may no longer be representative of natural populations at both genetic and phenotypic levels, and (ii) laboratory cultures may diverge from each other to such an extent as to no longer represent truly comparable genetic populations. Indeed, some researchers have questioned whether model organisms are truly representative, genetically and phenotypically, of natural populations of the same species (Gasch *et al*., 2016).

When a laboratory population is first established, it is usually started with a relatively small number of reproducing individuals, very likely resulting in a founder effect, i.e. a loss of genetic variation compared to the larger source population. This is due to a genetic bottleneck, or the fact that the small number of founder individuals do not carry the full range of gene variants (alleles) present in the source population (Barton and Charlesworth, 1984). Therefore, from the very beginning, a laboratory culture is likely to be different from the larger population from which it is derived. The information in Tables 1 and 2 confirms that laboratory cultures of the 2 helminth species considered here have often been founded by relatively few individuals. The number of founders was typically fewer than 10 worms for the cestode *H. diminuta*. Although a few hundred or a few thousand individual larvae were used for the nematode *H. polygyrus*, most of these larvae no doubt failed to establish in mice and develop into adult worms. In addition, these founders almost invariably came from another laboratory culture, which itself no doubt passed through a genetic bottleneck when it was established. In effect, this leads to sequential founder effects, compounding the reduction of genetic diversity relative to the ancestral natural population.

The goal of starting a laboratory culture was often to produce an inbred strain of parasites, by infecting only a few rodents in each generation and using eggs from only 1 or a few adult worms (i.e. each new generation produced by mostly full sibling parasites). However, loss of genetic variation is almost inevitable whatever the goal of the researchers starting a culture. Because laboratory facilities are limited in space and resources, the overall culture population will be kept to a small size following its establishment. Only so many cages housing host rats or mice can be accommodated, limiting the size of the adult worm population at any given time. The effective population size (i.e. the number of individuals that effectively participate in producing the next generation) is actually much smaller for 3 reasons. First, it is inevitable that out of all cysticercoids of *H. diminuta* and out of all infective larvae of *H. polygyrus* entering a definitive host, only a fraction will actually establish in the host's gut (Forrester and Neilson, 1973; Froelick *et al*., 2021). For example, infection of a mouse with 400 infective L3 larvae of *H. polygyrus* will typically lead to only about half of them establishing as adult worms (Johnston *et al*., 2015). The same is true for transmission of *H. diminuta* eggs to the beetle intermediate host (Dhakal *et al*., 2018). Therefore, only a subset of genotypes present at each stage of the life cycle makes it to the next stage. Secondly, cross-fertilization may not be the norm among individuals of the hermaphroditic cestode *H. diminuta* sharing the same rat host, as selfing appears to be a common and viable strategy in *Hymenolepis* spp. (Jones *et al*., 1971; Nakamura and Okamoto, 1993). Thirdly, among adult cestodes and nematodes in the gut of definitive hosts, typically the contribution of offspring to the next generation is extremely unequal, with only a few individual worms accounting for the vast majority of eggs released in host feces (e.g. Shostak and Dick, 1987; Szalai and Dick, 1989). Therefore, in both the founding population and the subsequent generations, the effective population sizes, from the perspective of genetic diversity, are relatively small in laboratory helminth cultures.

Small effective population sizes render laboratory helminth populations subject to random genetic drift, or changes in allele frequencies due to random chance events (e.g. occasional mortality of infected hosts). Some alleles can disappear from a small breeding population, whereas deleterious mutations can also accumulate over time in small populations, one of the key predictions of population genetics (Kimura *et al*., 1963). This so-called mutational load can result in a general reduction of fitness in small populations, as accumulating deleterious alleles which each have small effects on their own can have greater impacts collectively (Agrawal and Whitlock, 2012). Compounding these inevitable genetic processes, laboratory culture populations are not only small, but likely comprising genetically related and similar individuals. This can lead to inbreeding, and thus inbreeding depression, resulting from a greater likelihood of homozygosity for deleterious recessive alleles (Charlesworth and Willis, 2009). A recent genomic analysis of the nematode *H. polygyrus* based on specimens from a laboratory culture maintained for decades has indeed uncovered substantially greater homozygosity than in wild-sourced individuals (Stevens *et al*., 2023). Although often less pronounced in hermaphrodite species (Charlesworth and Willis, 2009), such as the cestode *H. diminuta*, inbreeding depression is yet another genetic process plaguing small populations, and likely to drive genetic and phenotypic divergence among laboratory and natural populations. Most cultures of *H. diminuta* and *H. polygyrus* have never been ‘refreshed’ genetically with the introduction of new individuals from an outside source (see Tables 1 and 2), therefore there has been no mitigation of the above processes.

It should be pointed out that these genetic issues are not restricted to the parasites, they also apply to their hosts. The laboratory hosts of both helminths, i.e. rats and mice, as well as the beetles used for *H. diminuta* (see Pointer *et al*., 2021), are themselves model species whose captive and laboratory-bred populations have undergone the same genetic processes (founder effect, genetic drift, etc.) as the 2 helminth species. Inbred strains of laboratory rats and mice have been produced decades ago and used widely in research ever since (Lindsey, 1979; Davisson and Linder, 2004); they are also used as hosts of the 2 helminth species in laboratory cultures.

Beyond stochastic fluctuations in allele frequencies, another important force acting on the genetic composition of laboratory culture populations is, of course, natural selection, or in this case artificial selection since organisms maintained in the laboratory are exposed to conditions vastly different from those experienced in nature. The often rapid responses of all kinds of organisms to novel selective pressures in captivity has been exploited by evolutionary biologists as an opportunity to study evolution as it happens (Kawecki *et al*., 2012). Many parasites have been shown to evolve new phenotypes within just a few generations under culture conditions. For example, serial passage experiments have shown that parasite virulence can evolve rapidly when transmission mode or transmission success is manipulated experimentally (Ebert, 1998). Similarly, the artificially high host densities in aquaculture can promote rapid evolution of both parasite virulence and compatibility with new host species (Nowak, 2007). A comparison between 2 laboratory strains of the trematode *Schistosoma mansoni*, both started with specimens collected from the same locality but 34 years apart, revealed differences in several traits, including virulence and egg production, indicating that the time (and thus the number of generations) spent in laboratory culture shapes the evolution of key phenotypic traits (Dias *et al*., 2023). In the cestode *Schistocephalus solidus*, only 4 generations of artificial selection were sufficient for the parasite to evolve significantly faster growth rates within its arthropod intermediate host (Benesh, 2023). Existing cultures of *H. diminuta* and *H. polygyrus* have been in continuous existence for many more generations than that (Tables 1 and 2), allowing ample time for selection to act on them. Thus, in the case of laboratory helminth cultures maintained for other purposes, artificial conditions exerting unnatural selective pressures can have unplanned, unanticipated and very rapid evolutionary consequences (see next section). The fact that these consequences can depend strongly on what alleles are available for selection in the population's gene pool will also contribute to phenotypic and genetic divergence among laboratory culture populations.

Not surprisingly, multiple studies have revealed some phenotypic (Kino and Kennedy, 1987) and genetic (Andrews *et al*., 1989; Dixon and Arai, 1991; Režábková *et al*., 2019) differences among laboratory cultures of the cestode *H. diminuta*, or between laboratory cultures and wild populations. However, genetic differences among laboratory cultures are generally modest and there is no evidence that they might represent distinct but cryptic species. For instance, Režábková *et al*. (2019) found only minor differences in nuclear and mitochondrial genes among specimens obtained from cultures in Europe and the United States, and between specimens from cultures and those from wild hosts, and concluded that they all belonged to a single genetic lineage.

In contrast, evidence of genetic divergence is somewhat stronger in the case of the nematode *H. polygyrus* (sensu lato). Whatever the exact wild source of the specimens that founded laboratory cultures (Behnke *et al*., 1991), there is significant genetic variation across the species' natural geographical range. A study of natural populations, based on mitochondrial DNA, uncovered 5 major lineages in Europe alone that have been isolated and diverged from each other for 1.5–2.5 million years (Nieberding *et al*., 2005). At the same time, evidence of genetic differences between *H. polygyrus* worms from wild hosts and worms from laboratory cultures in the United Kingdom was uncovered several years ago (Abu-Madi *et al*., 1994, 2000; Stevens *et al*., 2023), eventually leading to the proposal that they represent distinct species: *H. polygyrus* in wild hosts and *H. bakeri* in laboratory cultures (Cable *et al*., 2006; Musah-Eroje *et al*., 2023; Stevens *et al*., 2023). The nomenclatural change has been controversial (Behnke and Harris, 2010; Maizels *et al*., 2011) and it has not been adopted universally by all researchers, some preferring to treat laboratory worms as a subspecies, *H. polygyrus bakeri*. Regardless of these taxonomic issues, the many laboratory lineages differ functionally from wild populations, and probably also differ among each other.

For most practical purposes, it does not matter whether or not laboratory cultures are distinct genetically from their wild counterparts to the extent that they now represent different species within the same genus. As long as their biology is still representative of cestodes and nematodes in general, they remain extremely useful model organisms. The key word here is *representative*; in the next section, I argue that for certain traits, and thus for certain research areas, laboratory cultures of *H. diminuta* and *H. polygyrus* may no longer present characteristics that are fully representative of those of natural populations.

## Why genetics matters for research on model helminths

**Figure 7.**
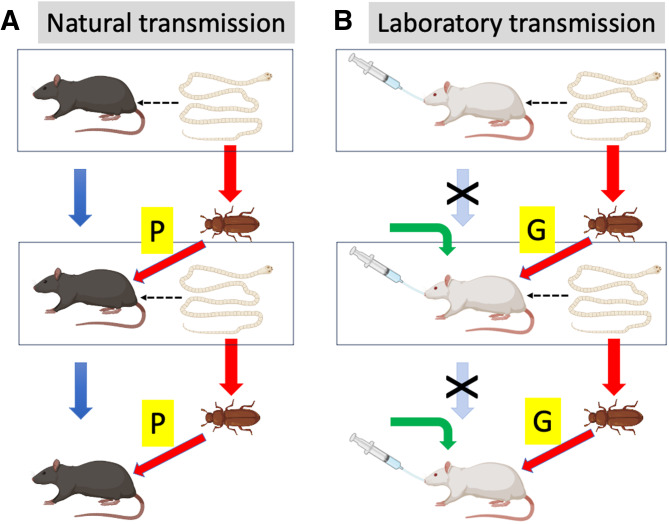
Trans-generational transmission of the cestode *Hymenolepis diminuta* under (A) natural and (B) laboratory culture conditions. In natural populations, in each generation (box), adult worms produce eggs that pass through beetle intermediate hosts and subsequently develop into the next generation (red arrows), while hosts also breed to give rise to the next host generation (blue arrows). However, in laboratory populations, the hosts used by the worms generally do not contribute to the next generation; instead, new naïve hosts are used in each generation (green arrows). Furthermore, in nature definitive hosts acquire parasites through predation (P) on infected beetles, whereas in laboratory cultures infection of the definitive hosts is achieved by dissecting cysticercoids out of beetles and then feeding them to definitive hosts by gavage (G). The figure uses icons from BioRender.com.

**Figure 8.**
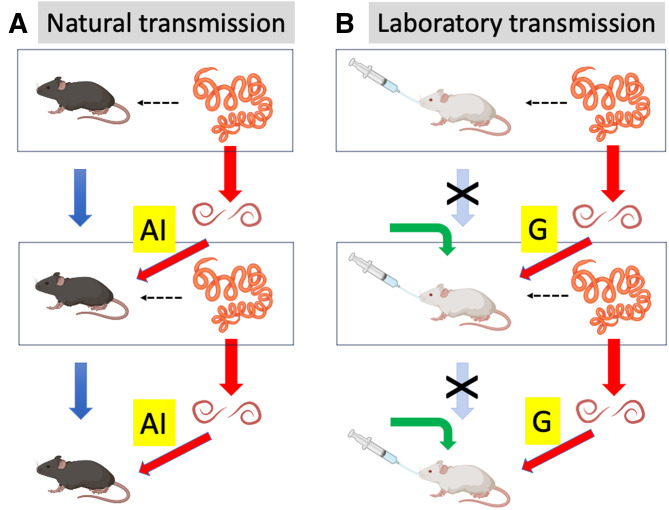
Trans-generational transmission of the nematode *Heligmosomoides polygyrus* under (A) natural and (B) laboratory culture conditions. In natural populations, in each generation (box), adult worms produce eggs that hatch into infective larvae and eventually develop into the next generation (red arrows), while hosts also breed to give rise to the next host generation (blue arrows). However, in laboratory populations, the hosts used by the worms generally do not contribute to the next generation; instead, new naïve hosts are used in each generation (green arrows). Furthermore, in nature hosts acquire parasites through accidental ingestion (AI), whereas in laboratory cultures infection is achieved by gavage (G). The figure uses icons from BioRender.com.

Whereas founder effects and genetic drift are associated with mostly neutral genetic variation, unplanned artificial selection is more likely to lead to phenotypic and functional changes in laboratory populations. Importantly, the unnatural conditions under which helminths are maintained and transmitted in laboratory cultures can exert selective pressures on several of the traits frequently investigated by researchers using these model organisms. In particular, the mode of transmission and resistance status of hosts of *H. diminuta* and *H. polygyrus* differ greatly between the natural and laboratory life cycles (Figs 7 and 8). Here, I consider some cases where unplanned evolution has likely caused evolutionary changes in parasite traits to the extent that the findings of many studies that used laboratory helminth cultures may need to be re-examined.

Host–parasite coevolution is the conceptual foundation underpinning our understanding of parasite virulence and the resulting pathology it induces on the one hand, and host resistance and tolerance on the other hand (Anderson and May, 1982; May and Anderson, 1983). In nature, an arms race is maintained generation after generation by the reciprocal selective pressures exerted by each antagonist on the other. The outcome has been the array of sophisticated immune defences seen in hosts and the elaborate counter-measures used by parasites. However, in the laboratory life cycles of *H. diminuta* and *H. polygyrus*, the host–parasite coevolutionary arms race no longer exists. Individual hosts that are experimentally infected with helminths are systematically removed from the breeding host population, and will generally not contribute offspring to the next generation. Each host infected by gavage comes from a naïve host population laboratory-bred in parasite-free conditions for multiple generations (Figs 7 and 8). There has been no selective pressure on that host's ancestors to retain efficient immune responses against helminths. There is no reason to expect its immune response to be fully representative of that of wild hosts. Similarly, beetles used as intermediate hosts for *H. diminuta* in laboratory cultures are themselves model organisms (Pointer *et al*., 2021) maintained in artificial parasite-free populations, with individuals utilized as hosts for the cestode also not used to perpetuate the captive beetle population. The way laboratory cultures of model helminth species are maintained leads to a complete disconnect between the evolutionary trajectories of hosts and parasites. The selective pressure exerted by parasitism on host defences is reduced to almost zero, whereas that exerted by host defences on parasite evasion mechanisms persists because the parasites that contribute to the next generation must still successfully infect a host. What, then, can we make of the findings of studies using these model species propagated across generations in a coevolutionary vacuum?

Studies on parasite immunogenicity and the intricacies of the host immune response have dominated research on the 2 model helminths, especially *H. polygyrus* (see Fig. 3). Yet the ability of parasites to cope with host immune defences depends on what selective pressures their recent ancestors have experienced. A recent serial passage experiment has demonstrated that after only 9 generations, *H. polygyrus* maintained in mice that were stimulated to generate a strong immune response evolved a stronger immunosuppressive ability than those maintained in control mice over the same period (Lippens *et al*., 2017; see also Su and Dobson, 1997). The possibility of selecting for parasites and creating strains that differ in how they elicit or evade immune responses is a great asset for researchers probing host immunology. However, it illustrates how laboratory cultures may also diverge from natural parasites. If the nematode evolves rapidly in response to the immune status of the hosts it encounters generation after generation, then we should expect worms in the typical laboratory culture setting (naïve hosts every generation) to gradually lose some of the mechanisms they use to escape host defences, especially if these mechanisms incur a fitness cost.

Evolution of parasite traits under laboratory conditions may also shape their virulence. In natural populations, a trade-off constrains the evolution of virulence: individual parasites that aggressively exploit host resources for their own reproduction face the prospect of a shorter reproductive period, as their host is more likely to die faster (Alizon *et al*., 2009). Virulent genotypes may be associated with fast growth and reproduction, as they use host resources at a fast rate regardless of its impact on the host, whereas less virulent genotypes may be more prudent in their exploitation of the host and take longer to reproduce and release eggs. However, in laboratory cultures, feces of infected hosts may be collected both shortly post-infection and many weeks later, and then used to infect the next host cohort. This allows both benign and virulent individuals to contribute genes to the next parasite generation in a manner that is not proportionate to their virulence. The procedures used to maintain laboratory cultures ensure transmission regardless of parasite traits; they can create a disconnect between what parasites do and their reproductive success, with evolutionary consequences for traits such as virulence.

Another common adaptive strategy of parasites is the ability of helminths transmitted by predation to modify the behaviour of their intermediate host in ways that increase the chances that the latter is captured and eaten by a definitive host, thus achieving parasite transmission. A few host–parasite systems have proven popular models to test hypotheses relating to parasite modification of intermediate host behaviour (Poulin and Maure, 2015). However, these model systems, such as the acanthocephalans *Polymorphus* spp. and *Pomphorhynchus* spp. and the amphipods *Gammarus* spp., rely on collections of naturally infected individuals from the field for each separate experiment, or the use of individuals following culture for no more than 1 or 2 generations in the laboratory. However, the cestode *H. diminuta* has been used in several studies of its effect on the behaviour of its beetle intermediate host, with varying results (Hurd and Fogo, 1991; Yan *et al*., 1994; Robb and Reid, 1996; Webster *et al*., 2000). A comparison of how transmission from intermediate to definitive host is achieved in natural *vs* laboratory conditions suggests that the use of *H. diminuta* from laboratory cultures may be completely inappropriate in this case (Fig. 7). In nature, rodents become infected when they prey on parasitized beetles. In this situation, how parasitized beetles behave relative to uninfected beetles plays a major role in determining the risk of predation they experience, and hence the probability of parasite transmission. This should select for parasite genotypes capable of altering beetle behaviour in ways that increase predation risk by rodents. In contrast, in laboratory conditions, transmission from beetle to rat is guaranteed regardless of how infected beetles behave: it is achieved by gavage, i.e. by researchers dissecting cysticercoids out of beetles, whatever the latter's behaviour, and force-feeding the cysticercoids to rats. There is no longer any benefit for the parasite associated with manipulating its host. The ability of the cestode to modify beetle behaviour becomes entirely uncoupled from its transmission success; the selective pressure to retain this ability has disappeared. The capacity to alter host behaviour is no doubt an evolutionary plastic trait. In a different cestode species, *S. solidus*, artificial selection in a laboratory population over just 3 generations resulted in significant changes in the manner and extent to which the cestode manipulated the behaviour of its arthropod intermediate host (Hafer-Hahmann, 2019). The number of generations over which *H. diminuta* cultures have been maintained (Table 1) is much higher, and undoubtedly sufficient for the ability of the cestodes to alter beetle behaviour to have decreased substantially. This would certainly be true in the case of selection acting directly against host manipulation abilities that impose a cost to the parasite, for instance, if they require the secretion of active substances. This is one more example of the potential evolutionary divergence that can occur between organisms maintained in laboratory cultures and their wild counterparts, casting some doubts over the general applicability of experimental results based on the former.

## Conclusions and recommendations

There is no doubt that laboratory cultures of parasites represent essential resources for research in parasitology, as they have in other areas of biology (Müller and Grossniklaus, 2010; Alfred and Baldwin, 2015). They provide a continuous supply of parasites of known genetic background, grown under standardized conditions, thus controlling for many of the sources of variance often plaguing the results of experiments on parasites of natural origins. Their use has opened doors to new research directions. For example, the fundamental knowledge of immunological processes gained from the use of *H. polygyrus* as a laboratory model has paved the way for the more recent transition to ‘wild immunology’ (Pedersen and Babayan, 2011), that is, the study of these processes in natural host–parasite systems. Model parasite species have played, and will continue to play, an important role in research on the impact and control of parasites in areas such as animal health and aquaculture (e.g. Hutson *et al*., 2018*a*, 2018*b*). Model parasites, in particular the 2 helminth species considered here, have also been invaluable tools for teaching parasitology to undergraduate students over the past few decades. However, as pointed out in this review, the maintenance of laboratory cultures of parasites under artificial conditions for multiple generations is not without risks: it can lead to the unplanned evolution of a genetically and phenotypically modified subspecies that may no longer be representative of natural populations. This is an unavoidable consequence of repeated serial passages through naïve hosts following unnatural modes of transmission. The same issues apply to laboratory cultures of parasites other than helminths (e.g. *Plasmodium* spp.; Claessens *et al*., 2017).

The use of model helminth species in laboratory cultures nevertheless remains one of the most powerful research tools available to parasitologists (Behnke *et al*., 2009; Vale and Duffy, 2021; Sulima-Celińska *et al*., 2022). Some simple recommendations emerge from the present synthesis to ensure their continued usefulness. First, all laboratory cultures should be ‘refreshed’ regularly with individuals from an external source. This has apparently occurred in only 4 of the 19 cultures for which data were obtained here (Tables 1 and 2), and in each of these few cases it occurred on a single occasion. Expanding the gene pool every few generations is essential to maintain the integrity of the laboratory ‘species’ and its genetic homogeneity among different cultures. This is achieved by dispersal and immigration in natural populations, processes that must be paralleled in laboratory cultures. Otherwise, helminth populations in different cultures may evolve along slightly divergent lines, due to random genetic drift exacerbating inter-culture variation associated with founder effects, making comparisons among studies based on different cultures more problematic.

Second, researchers using parasites from laboratory cultures should select the traits under study very carefully and be aware that they may have been modified by artificial selection across multiple generations. For instance, it may be inappropriate to use laboratory cultures of the cestode *H. diminuta* to study parasite manipulation of intermediate host behaviour, since the infection method used in culture has relaxed selective pressures to retain this strategy, and possibly even selected against it. In fact, whatever the parasite trait under study, the possibility of artificial selection under culture conditions demands that study results be subject to more nuanced interpretations, and their conclusions be extrapolated to natural populations with caution.

Third, however attractive research on laboratory models may be, especially when the research question requires established protocols or ready-made genomic tools, it cannot fully replace research on a broader range of parasite species if we are to achieve general answers to big questions. Recent evidence indicates that research funding goes preferentially to projects using established model organisms, at the expense of projects on non-traditional study organisms representing a broader taxonomic diversity (Farris, 2020). Research on both laboratory model helminths and an array of non-model species is necessary to attain a full understanding of the many issues of interest and relevance to parasitologists. Only a combination of these 2 approaches will allow us to achieve both depth and breadth of knowledge.

## Supporting information

Poulin supplementary material Poulin supplementary material

## Data Availability

All data used in this article are available in the Supplementary Material.

## Supplementary material

The supplementary material for this article can be found at https://doi.org/10.1017/S0031182023000963
